# Quantifying the Impact of COVID-19 on Telemedicine Utilization: Retrospective Observational Study

**DOI:** 10.2196/29880

**Published:** 2022-01-28

**Authors:** Emily Louise Vogt, Brandon M Welch, Brian E Bunnell, Janelle F Barrera, Samantha R Paige, Marisa Owens, Patricia Coffey, Nancy Diazgranados, David Goldman

**Affiliations:** 1 University of Michigan Medical School Ann Arbor, MI United States; 2 Office of the Clinical Director National Institute on Alcohol Abuse and Alcoholism National Institutes of Health Bethesda, MD United States; 3 Biomedical Informatics Center Medical University of South Carolina Charleston, SC United States; 4 Doxy.me Research Doxy.me, LLC Rochester, NY United States; 5 Department of Psychiatry and Behavioral Neurosciences University of South Florida Tampa, FL United States; 6 Clinical Center National Institutes of Health Bethesda, MD United States; 7 Lab of Neurogenetics National Institute on Alcohol Abuse and Alcoholism National Institutes of Health Rockville, MD United States

**Keywords:** telemedicine, COVID-19, utilization, impact, retrospective, observational, trend, telehealth, health policy, policy

## Abstract

**Background:**

While telemedicine has been expanding over the past decade, the COVID-19–related restrictions regarding in-person care have led to unprecedented levels of telemedicine utilization. To the authors’ knowledge, no studies to date have quantitatively analyzed both national and regional trends in telemedicine utilization during the pandemic, both of which have key implications for informing health policy.

**Objective:**

This study aimed to investigate how trends in telemedicine utilization changed across the course of the COVID-19 pandemic.

**Methods:**

Using data from doxy.me, the largest free telemedicine platform, and the NIH (National Institutes of Health) Clinical Center, the largest clinical research hospital in the United States, we assessed changes in total telemedicine minutes, new provider registrations, monthly sessions, and average session length from March to November 2020. We also conducted a state-level analysis of how telemedicine expansion differed by region.

**Results:**

National telemedicine utilization peaked in April 2020 at 291 million minutes and stabilized at 200 to 220 million monthly minutes from May to November 2020. Surges were strongest in New England and weakest in the South and West. Greater telemedicine expansion during the COVID-19 pandemic was geographically associated with fewer COVID-19 cases per capita. The nature of telemedicine visits also changed, as the average monthly visits per provider doubled and the average visit length decreased by 60%.

**Conclusions:**

The COVID-19 pandemic led to an abrupt and subsequently sustained uptick in telemedicine utilization. Regional and institute-level differences in telemedicine utilization should be further investigated to inform policy and procedures for sustaining meaningful telemedicine use in clinical practice.

## Introduction

In the past three decades, telemedicine—defined by the National Institutes of Health (NIH) as the use of telecommunication and technological services to provide and support medical care at a distance—has been widely adopted by health care providers and systems across the world [1,2]. Telemedicine can include synchronous modalities (ie, real-time audio or audiovisual interaction), asynchronous modalities (ie, messages or images exchanged via a patient portal), or remote patient monitoring. However, synchronous audiovisual communication attempts to closely replicate ordinary patient-provider or provider-provider interaction to the maximum extent possible given the limitations in audiovisual communication caused by vision or hearing impairments, both of which are prevalent among patients and providers [3,4].

The main advantages of telemedicine include improved accessibility of care, particularly for rural and underserved communities; flexibility of scheduling; greater continuity of care; reduced cost of care in certain situations; and enhanced collaboration between medical providers [5-7]. The information technology revolution, including the rapid expansion of electronic health record (EHR) usage and sharing, has accelerated the expansion of telemedicine, with more than 60% of US health care systems and 40% to 50% of US hospitals employing some form of telehealth [5]. Due to the COVID-19 pandemic in 2020, telemedicine abruptly became the safer form of care in many cases and the only practically allowable form in others.

Care delivery using telemedicine, however, is not without behind-the-scenes complexities, including coordination across EHRs, patient portals, e-prescribing platforms, other scheduling- or monitoring-related applications; access to secure, effective audiovisual communication software; and acquisition of sufficient computer hardware and high-speed internet. Due to these nuances of telemedicine, a large and growing body of research has emerged to study telemedicine’s efficacy across a diverse set of care delivery settings, patient populations, medical specialties, and geographic regions.

Telemedicine research has been an area of broad interest and development since the 1990s; in both 2018 and 2019, there were nearly 3000 publications each year related to telemedicine or telehealth [8]. These studies, largely in behavioral health-related settings, have demonstrated that the consumer experience is equivalent or superior to in-person encounters across a range of diagnoses, patient populations, and health care settings [7,9-11]. Moreover, under certain circumstances telemedicine is more cost-effective than in-person care and can allow health care entities to provide health care where it may otherwise be unavailable, either due to physical distance between the provider and patient or distance between a group of patients who can interact remotely but not in the same physical setting [7,12-14]. However, there has historically been a gap between the increasing research interest in telemedicine and its broad-scale implementation and acceptance by health systems due to reimbursement limitations, technical barriers, physician attitudes, and lack of physician education [15,16]. Physician skills may themselves need to be adapted and optimized for virtual care settings [15,17,18].

During the COVID-19 pandemic, telemedicine has become vital, or mandatory, for many purposes for which it was formerly convenient or optional. Due to the serious and ongoing safety risks associated with the spread of COVID-19 in in-person health care settings, widespread telemedicine adoption has become a necessary substitute for everything from routine health maintenance visits or exams to COVID-19–related issues. In March 2020 alone, the Cleveland Clinic reported 60,000 telehealth visits, a 1700% increase from the previous monthly average [19]. Similarly, a retrospective analysis of January to March 2020 from the Centers for Disease Control and Prevention (CDC) showed a 154% increase in telehealth visits in the last week of March, with COVID-19–related visits comprising 15.2% of these visits [20]. More recent data from the Veterans Affairs health administration indicated that trends have persisted well into the later stages of the pandemic, with a tally of more than 300 million virtual visits in the month of June 2020 alone [21]. Moreover, a recent survey of multispecialty physicians revealed that although only 12% of those surveyed had used telemedicine prior to the pandemic, 91% planned to continue offering telemedicine services following COVID-19 [22]. Thus, understanding the nuances of these trends in telemedicine usage has significant implications for not only the COVID-19 era, but for the future of health care [1,23-25].

While many patients and physicians initially resorted to familiar platforms such as FaceTime for virtual medical appointments, it was quickly recognized that these platforms did not assure security, privacy, or quality, which pushed many health care providers and systems to consider using existing telemedicine platforms. Doxy.me is a national telemedicine platform launched in 2013 primarily to serve mental health providers that has since been scaled to cover all types of medical specialties. While there are other commercial telemedicine platforms in the rapidly expanding telehealth industry, doxy.me is the largest platform that is both free and HIPAA (Health Insurance Portability and Accountability Act)-compliant, making it a popular choice for providers looking for a swift transition to virtual care amidst the COVID-19 pandemic [26-28]. Even as other telemedicine platforms emerged, trends in the usage of doxy.me can therefore reveal national changes in the use of telemedicine occurring in response to the pandemic due to its widespread, national utilization.

In contrast, some institutions like the NIH Clinical Center, known as “America’s research hospital” [29], elected to develop their own internal telemedicine programs. While these programs took longer to jumpstart given that they had not been established prior to the pandemic, they allowed institutions like the NIH Clinical Center to ensure full integration with their existing hospital and research infrastructure, as well as the sustainability of the program beyond the pandemic. The aim of this study was to comparatively analyze trends in telemedicine utilization during COVID-19 from both doxy.me’s national platform and the NIH Clinical Center’s program to better quantify how and where the COVID-19 pandemic has most influenced telemedicine usage. Synchronous audiovisual visits, and the ancillary factors that enable them, were the focus of this study.

## Methods

### Study Design

We conducted a retrospective observational study of trends in telemedicine utilization from two different stakeholders: doxy.me, a national platform that has been supporting telemedicine encounters prior to COVID-19; and the NIH Clinical Center, the nation’s largest clinical research hospital, which developed a telemedicine program in response to COVID-19. Doxy.me’s workflow is designed to be as simple as possible while still being familiar to both patients and doctors, with a check-in feature, a waiting room, and a patient queue. The virtual platform attempts to greet patients with a routine that feels familiar to the clinical experience. No patient information is stored by doxy.me, all calls are encrypted end to end by default, and the platform is built on top of an open-source standard for real-time communication over the internet. The platform is built to only operate within trusted web browsers provided by Mozilla, Apple, Google, and Microsoft, who update their products on a rolling cycle every 6 to 9 months to ensure they are patched and up to date. Our study analyzed doxy.me usage from January to November 2020, the period in which the pandemic and, consequently, the need for virtual encounters accelerated most rapidly.

The NIH Clinical Center’s telemedicine program is markedly different from doxy.me, being institution-specific in scope and having been developed directly in response to COVID-19 for secure videoconferencing with past and present patients at the NIH Clinical Center. It is based on Microsoft Teams (Microsoft Corp), a platform compliant with the NIH’s privacy and security policies. Information about the NIH Clinical Center’s program was provided to individual NIH institutes via Medical Executive Committee meetings and a web-based telehealth resources section. This program was launched in April 2020, and this analysis encompasses all telehealth visits between April and November 2020 at the NIH Clinical Center.

Both doxy.me and the NIH Clinical Center track metrics for quality assessment and quality improvement purposes. Doxy.me tracks registered providers, sessions, and minutes as a measure of growth, and conducts annual risk assessments and updates per HIPAA and Health Information and Technology for Economic and Clinical Health (HITECH) policies accordingly. Updates include vulnerability and patching updates, backup and business continuity plans, encryption of data stored at rest or transfer per the recommendations of the National Institute of Standards and Technology, and regular access and error log audits via an intrusion detection system. Notably, any provider using the platform can generate a Business Associate Agreement signed by doxy.me, to assist in maintaining HIPAA/HITECH compliance across the board. Doxy.me also allows for the storage of emergency forms with the primary provider’s contact information for each patient. In contrast, the NIH Clinical Center’s data tracking includes linkage of all data contained in the EHR. To initiate an NIH Clinical Center telehealth encounter, care teams enter an electronic appointment request in the EHR. The Health Information Management Department conducts a documentation review for each patient encounter. Completed, documented appointments are marked as “arrived” and subsequently counted as telehealth visits.

### Statistical Analysis

Trend analysis was performed on both data sets. For doxy.me data, regression analysis was performed to illuminate state-level trends in telemedicine versus COVID-19 case rates. State-by-state COVID-19 case rates were derived from the CDC’s database [30]. Additionally, US census data were used to normalize levels of telemedicine utilization and COVID-19 cases across different states. Data analysis and graphical depictions were performed using Microsoft Office (Microsoft Corp).

## Results

### Nationwide Telemedicine Trends

**Figure 1:**
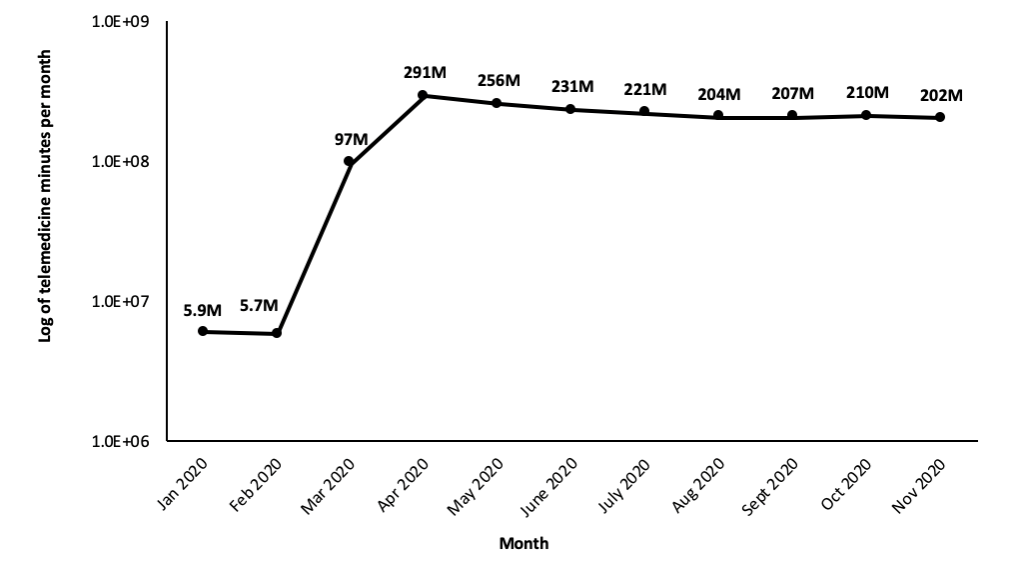
The total number of session minutes per month logged on the doxy.me platform from January to November 2020. These data demonstrate that while monthly minutes peaked in April at 291 million minutes, the high level of utilization largely plateaued from May to November.

**Figure 2:**
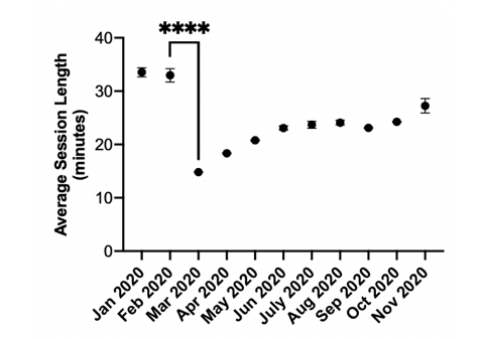
The average session length per doxy.me provider per month from January to November 2020. These results elucidate the dramatic decrease in telemedicine session length (*P*<.001) at the onset of the pandemic, from February to March. From April to November, the average telemedicine session length normalized back to pre–COVID-19 levels with the average session length during November not significantly lower than in January (*P*=.10). Asterisk indicates a statistically significant difference.

Doxy.me’s telemedicine volume surged from just over 5.5 million monthly minutes in February 2020 to more than 89 million monthly minutes in March 2020 (Figure 1), a 29-fold increase compared to usage data from March 2019. Increases in the number of telemedicine sessions per provider were also observed, as was a highly significant decrease in the average length of telemedicine sessions during March and April 2020 compared to prepandemic times (Figure 2). This finding indicates a change in the average telemedicine encounter, the nature of which is not fully understood.

Doxy.me’s provider registrations—tracking the number of new providers signing up on their platform—revealed that the intensified use of telemedicine was mainly driven by new users. March yielded the greatest number of new provider registrations at 299,324, a more than 3-fold jump in doxy.me’s total provider base in just 1 month. Predictably, this uptick in provider registrations occurred in tandem with the initial declaration of COVID-19 as a global health emergency by the World Health Organization and with stay-at-home directives in the United States. However, since May 2020, new provider registrations have stabilized at a much lower level, fluctuating between 12,000 and 19,000 new registrations per month.

Similarly, total minutes of telemedicine utilization accelerated rapidly in March and April 2020 but then stabilized at a new, markedly higher (48×) level as compared to prepandemic times, approaching 300 million minutes/month in April and settling at just over 200 million minutes/month (Figure 1).

### Regional Telemedicine Trends

**Figure 3:**
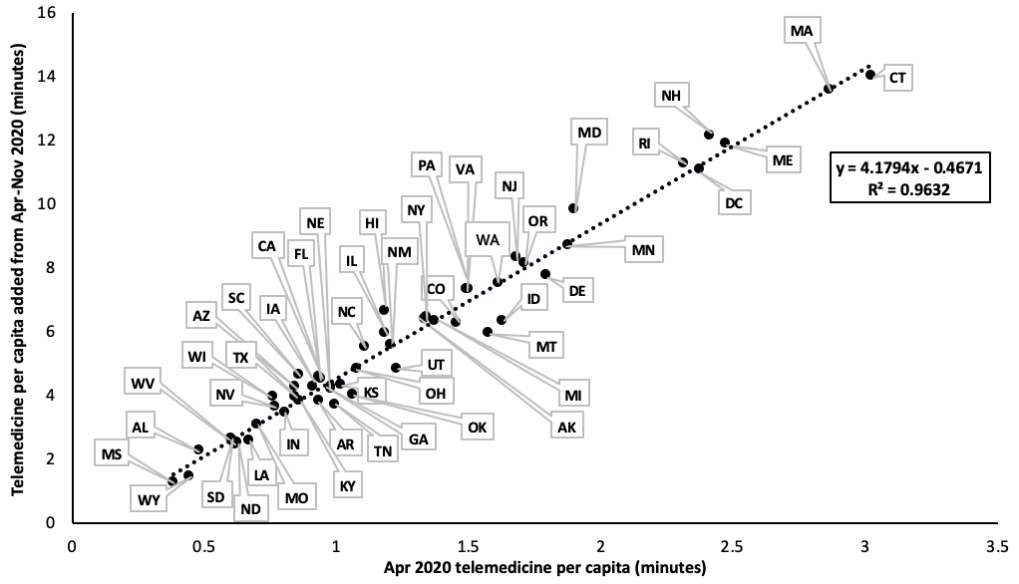
State-by-state comparison of April 2020 telemedicine minutes per capita versus telemedicine per capita added during the onset of the COVID-19 pandemic (measured as April through November 2020). Linear regression analysis shows a strong, positive association (m=4.18; R2=0.96) between states that had more telemedicine capacity in April and greater overall telemedicine expansion during COVID-19. Note: Vermont was excluded from this graph as it was an outlier at (4.66, 16.34).

**Figure 4:**
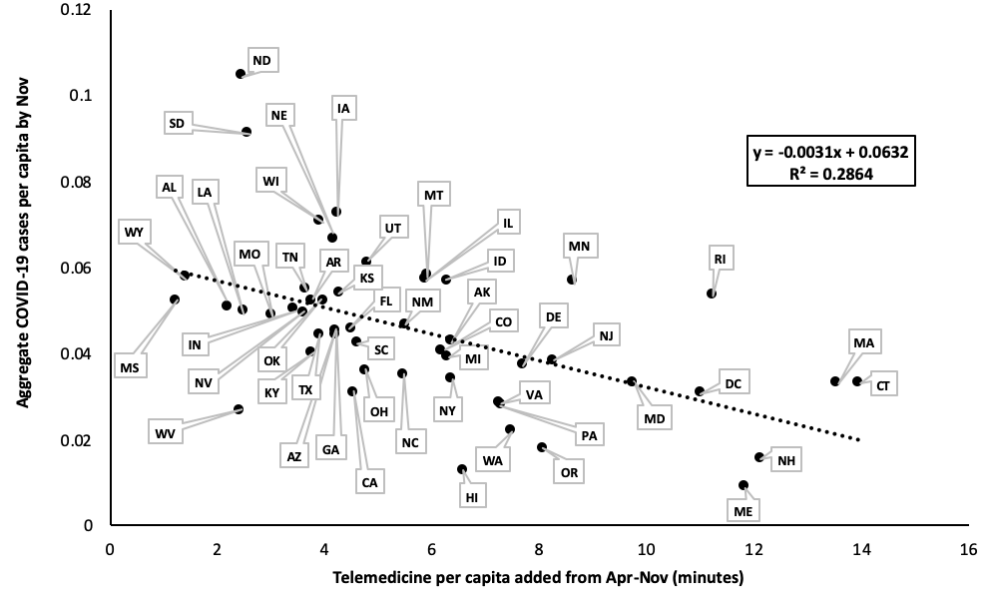
State-by-state comparison of telemedicine per capita added during the onset of the COVID-19 pandemic (measured as April through November 2020) versus aggregate COVID-19 cases per capita through November 2020. Linear regression analysis shows a moderately negative relationship (m=–0.0031, R2=0.29), indicating that greater telemedicine expansion was somewhat associated with fewer aggregate COVID-19 cases per capita. Note: Vermont was excluded from this graph as it was an outlier at (19.61, 0.0067).

Telemedicine utilization across states and over the course of the pandemic revealed that the surge in the utilization of telemedicine was highly correlated with the earlier expansion of telemedicine service capacity in certain states (m=4.18; *R*^2^=0.96), presumably driven by discrepancies in the supply of existing telemedicine providers in certain states versus others (Figure 3). In other words, states with more providers who were already licensed and practicing telemedicine prior to the pandemic had a greater capacity for offering telemedicine services at the onset of the pandemic compared to states with few to no trained telemedicine providers. Surprisingly, states with the greatest expansion of telemedicine provider registrations also tended to have lower aggregate rates of COVID-19 cases per capita (m=–0.0031; *R*^2^=0.29) (Figure 4). Regional trends in the expansion of telemedicine were evident (Figures 3 and 4). New England (including Massachusetts, Connecticut, New Hampshire, Maine, Rhode Island, and Vermont; see note in Figure 3 for Vermont) had the strongest telemedicine expansion early in the pandemic, both in more rural and metropolitan areas. These New England states also had the lowest rates of COVID-19 per capita by November (see Figures 3 and 4). The Mid-Atlantic states (including New York, Pennsylvania, New Jersey, Delaware, Maryland, Virginia, and the District of Columbia) experienced a moderate expansion of telemedicine between April and November, and moderate to low aggregate COVID-19 cases per capita by November, despite initial COVID-19 hotspots in New York City and New Jersey (Figures 3 and 4).

The southern and northwestern states, most notably Mississippi, Alabama, Wyoming, and the Dakotas, had the slowest expansion of telemedicine possibly because of a delay in the upswing in cases and a lag in the implementation of social distancing measures and mask mandates (Figure 3). These states also logged some of the highest rates of COVID-19 cases per capita by November (Figure 4). Thus, it seems that the expansion of telemedicine services across the country seemed to cluster with states that also had a stricter mask mandate and social distancing policies, suggesting the potential role of regional politics in shaping telemedicine adoption.

### NIH Clinical Center Program Outcomes

**Figure 5:**
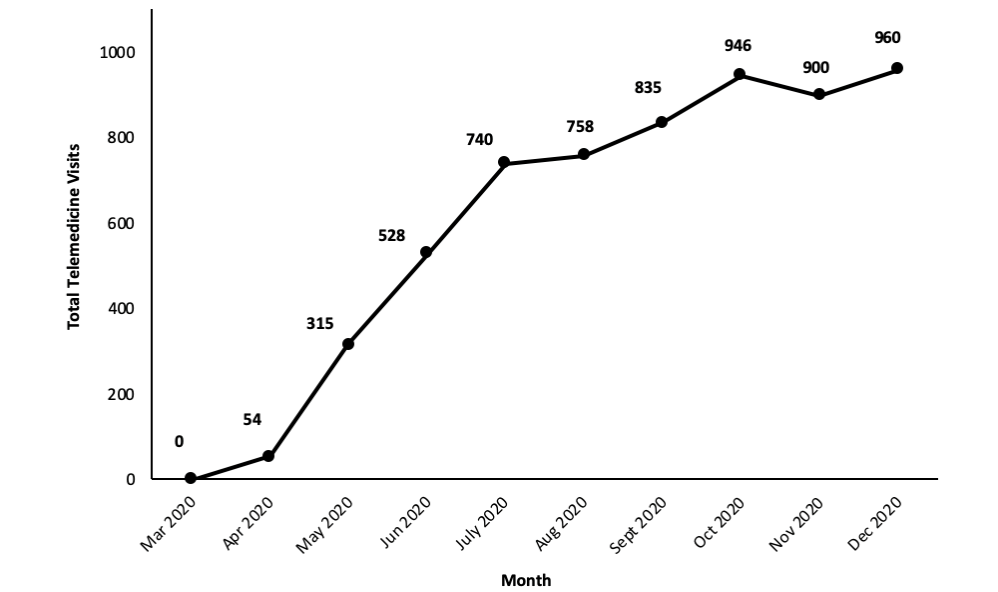
Increase in telemedicine visits at the NIH Clinical Center between its initiation on April 15 and December 2020. Trends show that there was a steadily increasing number of monthly visits since its initiation, with November as the only month that did not have more visits than the previous one. In just 7 months, the program tallied more than 6000 total visits.

NIH Clinical Center data indicate that telemedicine rapidly became a useful tool for continuing clinical research efforts at a distance and has continued to accelerate in terms of utilization. Importantly, the NIH Clinical Center was closed to admissions except for COVID-19 cases and medical emergencies from April to June 2020, but the usage of telemedicine has continued to accelerate after the NIH Clinical Center was reopened to patients in most research studies. Although the NIH Clinical Center’s telemedicine program has only existed since late April, it has already logged nearly 3000 visits across 15 different institutes (Figure 5). Most of these visits were between a remote patient and provider; however, some visits have been between patients at the NIH Clinical Center and specialists or other providers who are teleworking due to COVID-19 staffing restrictions. In addition, patient group meetings and staff meetings, including clinical rounds on inpatients, are conducted remotely or with a large remote component even though they are beyond the scope of the data presented here.

## Discussion

### Principal Results

In summary, this study demonstrated that telemedicine expanded by more than 50-fold its previous level at the onset of the COVID-19 pandemic and has continued to maintain this high level of utilization across subsequent months, particularly within states in New England and the Mid-Atlantic. The rapid expansion of telemedicine during the COVID-19 has been widely appreciated and qualitatively described [20,31-34]. Here, we have added a quantitative analysis of trends in telemedicine demonstrating a sudden expansion (ie, over only 2 months) at a very large magnitude expansion, probably representing more than a 50-fold increase in telemedicine on a nationwide basis. In this expansion, an earlier adoption of telemedicine was associated with a greater magnitude of increase. There is strong evidence that, on a national level, the use of telemedicine has plateaued at a formerly inconceivable 200 million minutes/month on the doxy.me platform alone. Furthermore, there is evidence that the nature of the typical telemedicine visit has changed during the pandemic, now averaging only about 15 minutes instead of twice that, and with twice as many visits per provider per day. This change in pattern of use seems to reflect the fact that much of the use of telemedicine previously was for behavioral health purposes, rather than the broader clinical applications that have defined the COVID-19 era.

Doxy.me’s telemedicine utilization indexes the private telemedicine market—a space that is likely to continue to grow and, through its growth, shift the landscape of health care in the years to come. Data from doxy.me’s platform demonstrates that telemedicine suddenly became a critical tool for remote care during March and April 2020, when hospitals were overwhelmed with patients with COVID-19 and other facilities were largely shut down. Comparing March 2020 to March 2019, there was a 48-fold increase in the number of sessions on this platform, with it being one of the most widely used platforms before and after the beginning of the pandemic. Interestingly, the unprecedented surge in telemedicine usage has been relatively sustained since it peaked at 291 million monthly minutes in April, with the months of May to November 2020 still averaging 200 to 220 million monthly minutes each. This finding suggests that telemedicine utilization was not strictly determined by fluctuating COVID-19 case rates and stay-at-home orders; rather, telemedicine has sustained its role in serving both patients with and without COVID-19, a role that will likely continue in a postpandemic era.

Regional differences in telemedicine utilization identify geographically clustered states that either had greater (in the case of New England and the Mid-Atlantic) or lesser (in the case of southern and western states) degrees of telemedicine implementation and utilization during COVID-19 (Figures 3 and 4). One explanation for this finding is that northeastern states were forced to adopt telemedicine earlier (Figure 3). Additionally, the moderately negative association between telemedicine expansion and aggregate COVID-19 cases demonstrates that the earlier expansion of telemedicine capacity meant there were fewer in-person, health-related appointments in these states, which helped to contain the spread of COVID-19 and keep case rates lower than states without the same degree of telemedicine utilization. This association demonstrates the efficacy of telemedicine as a public health tool, especially in pandemic-related situations. However, it is also likely that the earlier adoption of telemedicine in certain states was attributable to other factors, including differences in health care infrastructure, reimbursement policies, and licensing restrictions. More specifically, we would like to note that while many insurance companies had waived previous telemedicine-related reimbursement restrictions in light of the need for virtual care during the pandemic [35], these cost- and insurance-related barriers can significantly skew who is able to access telemedicine or other means of virtual care delivery [15]. While it is beyond the scope of this paper to explore these other factors, our results paint a picture of how telemedicine has evolved throughout the COVID-19 pandemic on a macroscopic level and provides a basis for speculating how it may continue to evolve, in tandem with necessary policy changes to make telemedicine more accessible to all patients and caregivers.

In contrast, the NIH Clinical Center’s telemedicine program provides an alternative perspective on how a program that emerged in response to COVID-19 could still impact daily clinical research operations during both lockdown and return-to-work phases. The results from the NIH program indicate that there is an accelerating role for the use of telemedicine for clinical researchers to follow up with patients, schedule remote study visits, and provide virtual support to groups of patients. Given the sheer number of research protocols suspended or halted during COVID-19, this telemedicine program has evolved to support nearly 1000 patient visits each month, a number that represents most active clinical research visits. While the majority of telemedicine visits in the initial weeks of the program were within the National Cancer Institute, there has been a marked uptick in the proportion of visits for inpatient mental health units including those of the National Institute on Alcohol Abuse and Alcoholism (NIAAA) and the National Institute of Mental Health (NIMH) from June to August. This shift can largely be attributed to the fact the NIAAA and NIMH inpatient units were closed at the onset of the pandemic and did not reopen until the summer; however, it is also interesting to consider whether some of the increases in telemedicine utilization by these units may be indicative of broader increases in mental health needs following the trauma and anxiety caused by COVID-19.

### Limitations

While both the doxy.me platform and the NIH Clinical Center program provide a broad snapshot of changes in telemedicine utilization and program development during the COVID-19 pandemic, they are by no means all-inclusive of the changes happening across all health systems and platforms. Additionally, while this analysis was focused on quantitative trends in national and regional differences in telemedicine utilization, we did not have access to meaningful data to analyze patient- and provider-level differences in access to telemedicine during COVID-19, which may be important for further shaping policy efforts. Future studies should aim to understand how these patient- and provider-level factors, including provider specialty, provider age, patient age, and patient socioeconomic status, may influence access to telemedicine-based care.

### Conclusions

Overall, data from both these programs provide a quantitative lens for examining how trends in telemedicine have changed in response to COVID-19, with meaningful implications for local and national health care policy. Telemedicine utilization increased more than 48-fold in the first year of the pandemic, most notably in New England and the Mid-Atlantic. While telemedicine has provided significant bandwidth and has played an important role in covering remote care delivery needs, there are some apparent limitations of telemedicine as the sole option for care. Many specialties are limited in terms of the care they can provide virtually. Additionally, data from this analysis revealed that there were marked decreases in the amount of time providers spent per session in virtual appointments, whether attributable to telemedicine, the demands of the pandemic, or both. It is highly unlikely that telemedicine will displace in-person care efforts in any medical specialty. However, this study illustrates some important considerations as we evolve to a more hybrid model of virtual and in-person care, including privacy- and security-related nuances, regional differences, and clinical setting considerations. Even beyond the COVID-19 pandemic, telemedicine will continue to shape the evolving nature of health care delivery and hold critical importance for increasing access to health care.

## Abbreviations

CDC: Centers for Disease Control and Prevention
EHR: electronic health record
HIPAA: Health Insurance Portability and Accountability Act
HITECH: Health Information Technology for Economic and Clinical Health
NIAAA: National Institute on Alcohol Abuse and Alcoholism
NIH: National Institutes of Health
NIMH: National Institute of Mental Health
